# Supplementary material for: Particulate Matter Increases the Severity of Bleomycin-Induced Pulmonary Fibrosis through KC-Mediated Neutrophil Chemotaxis
Source: Int J Mol Sci. 2019 Dec 28;21(1):227. doi: 10.3390/ijms21010227 (PMC6981983; doi:10.3390/ijms21010227)
Supplement: Supplementary file 1 [file ijms-21-00227-s001.pdf]

Supplementary Figures

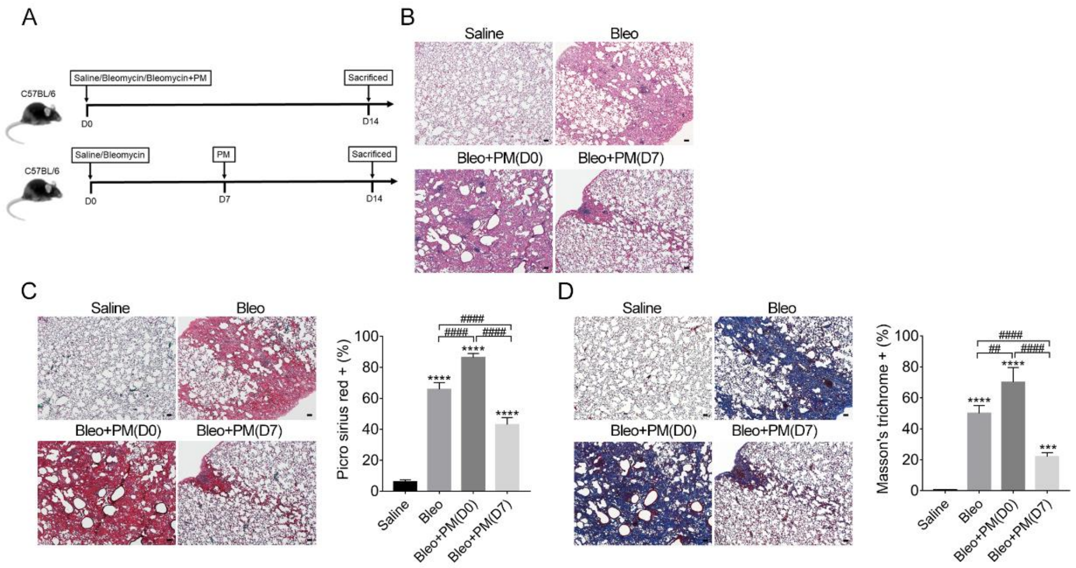

Figure S1. Compare the lung section of PM instillation with bleomycin at day 0 with instillation at day 7 after bleomycin administration. (**A**) C57BL/6J mice received 2 U/kg bleomycin (Bleo), 2 U/kg bleomycin plus 200 μg articulate matter at day 0 (Bleo+PM D0) or 2 U/kg bleomycin at day 0 plus 200 μg articulate matter at day 7 (Bleo+PM D7). Mice were sacrificed with pentobarbital overdose on day 14 for (**B**) H&E staining (**C**) Picro Sirius red, and (**D**) Masson's trichrome (40×). (Scale bar: 200 μm) (C)(D) Quantification data are expressed as the mean ± SD of four independent animals. (Scale bar: 200 μm) ***$p < 0.005$, ****$p < 0.001$ versus Saline as determined by One-Way ANOVA. ##$p < 0.01$, ####$p < 0.001$ as determined by One-Way ANOVA.

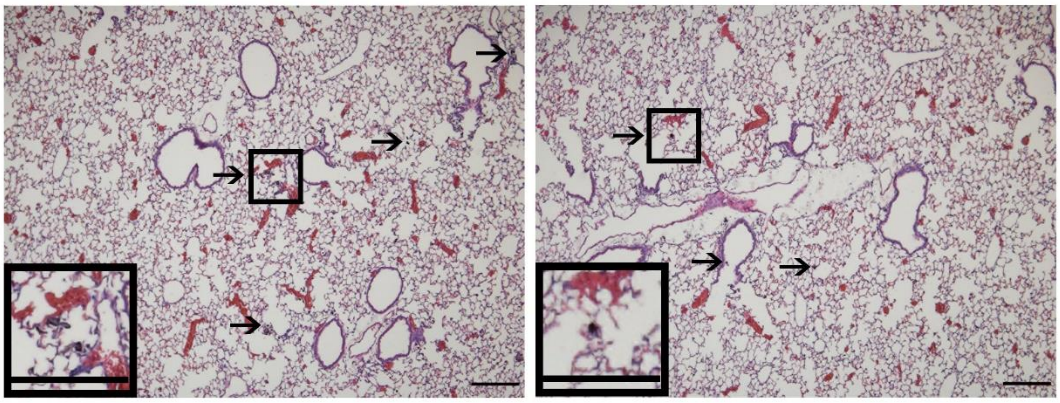

Figure S2. The distribution of the particles in the lung of mice after 14 days of receiving PM. C57BL/6J mice received 200 μg articulate matter at day and sacrificed with pentobarbital overdose on day 14 for H&E staining (40×). (Scale bar: 200 μm).

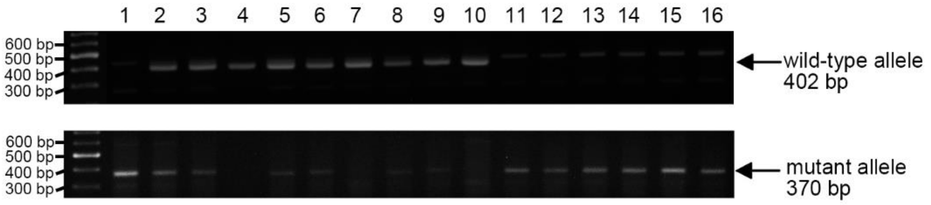

Figure S3. The genotyping of KC knockout mice. Genomic DNA was isolated from mouse tail and amplified by PCR. KC knock out mice were identified using different paring primers. The wild-type allele has 402 bp but the mutant allele has 370 bp.

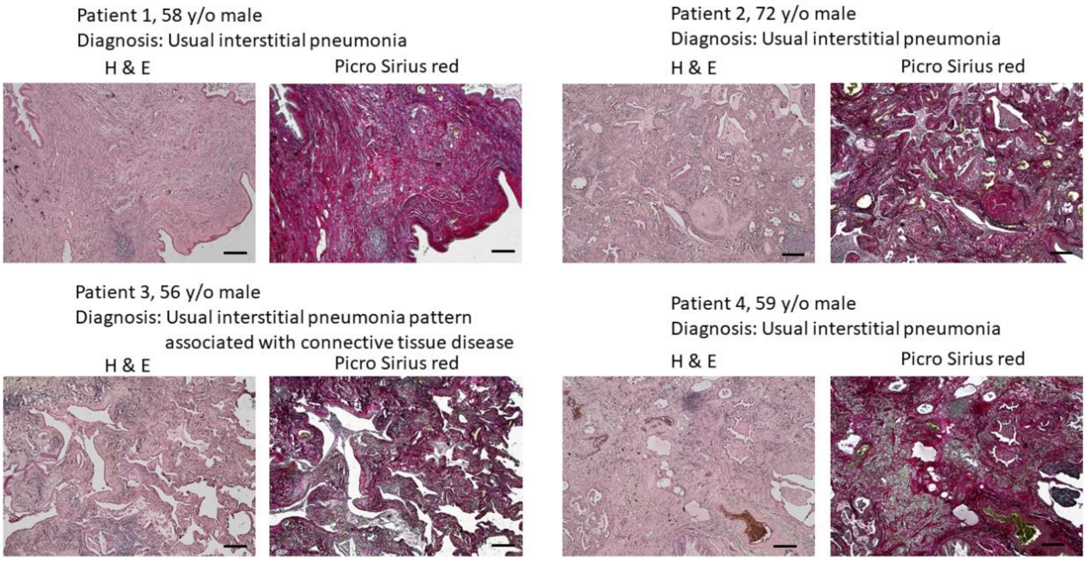

Figure 4. The demographic data and pictures of four human pulmonary fibrotic tissue blocks. Slices from patients who were diagnosed as usual interstitial pneumonia were stained by H&E and Picro Sirius red (40×). (Scale bar: 200 μm) .
